# Draft Genome Sequences and Complete Sequences of Two Plasmids of Escherichia coli Strain BOq 01, a Multiple-Antibiotic-Resistant Strain Isolated from a Poultry Farm in Mexico

**DOI:** 10.1128/MRA.01104-18

**Published:** 2018-11-21

**Authors:** Armando Hernández-Mendoza, Daniel Rivera Mendoza, Abimael Moran-Vazquez, Edgar Dantán-González

**Affiliations:** a Laboratorio de Estudios Ecogenómicos, Centro de Investigación en Biotecnología, Universidad Autónoma del Estado de Morelos, Cuernavaca, Morelos, Mexico; b Centro de Investigación en Dinámica Celular, Instituto de Investigación en Ciencias Básicas y Aplicadas, Universidad Autónoma del Estado de Morelos, Cuernavaca, Morelos, Mexico; University of California, Riverside

## Abstract

We report here the draft genome sequence of Escherichia coli strain BOq 01, a bacterium isolated from a poultry farm; the genome includes two plasmids conferring antibiotic resistances. This bacterium has a GC content of 50.89% and a genome size of 4.6 Mb.

## ANNOUNCEMENT

Many plasmids are present in different bacteria, and some of them carry multiple virulence and antibiotic resistance genes (1). Although plasmids impose a fitness cost on their hosts, evidence suggests that some beneficial traits, such as antimicrobial resistance, among others, would assist in maintaining their presence in the bacteria (2). The use of antibiotics in farms is a strong artificial selection for plasmids in bacteria. We sequenced the genome of Escherichia coli strain BOq 01, which shows resistance to six antibiotics (ampicillin, kanamycin, chloramphenicol, streptomycin, tetracycline, and nalidixic acid), prepared according to the 2017 CLSI standard (3); the bacterium was isolated from a chicken fecal sample in Tehuacán, Puebla, Mexico, and grown in solid rich medium. This strain has two plasmids, pBOQ-IncQ (4 kb) and pBOQ-95LK (95 kb), that help explain the resistance to at least two antibiotics (kanamycin and streptomycin).

A culture of BOq 01 was grown at 30°C with shaking at 250 rpm in Luria-Bertani broth for 12 h, genomic DNA was obtained using the AxyPrep bacterial genomic DNA miniprep kit (Axygen), and 5 μg of genomic DNA was used for whole-genome DNA sequencing using the Illumina HiSeq platform (2 × 300-bp paired-end) system. We obtained 1,352,179 paired-end reads that represent 180× coverage. The read quality was analyzed with FastQC (4), and quality-based trimming was performed with a DynamicTrim (SolexaQA++) (5) perl script with a minimum phred score of 20. The Illumina reads were assembled *de novo* using the SPAdes program (version 3.9.0), with *k*-mer odd values from 21 to 71, generating 74 contigs. Contigs with a length less than 500 bp were discarded and the remaining ones used for a multidraft-based analysis using three Escherichia coli genomes [those of strains ATCC 8739, BL21(DE3), and MG1655] through the MeDuSa scaffolder (6). The final version of the genome embeds 12 scaffolds, containing 4,601,531 bp, with a general GC content of 50.89%; 2 of the scaffolds are the plasmids pBOQ-IncQ (Scaffold_10_pBOQ-IncQ; 4,640 bp and 59% GC content) and pBOQ-95LK (Scaffold_12_pBOQ-95LK; 95,980 bp and 47.8% GC content).

The 12 contigs were analyzed on the RAST (version 2.0) server (7), and 4,071 open reading frames (ORFs) were identified, including 4,019 coding sequences (CDS) and 52 RNAs. The scaffolds were analyzed by RNAmmer (version 1.2) (8) and identified one instance of the 5S-23S-16S rRNA cluster in Scaffold_1, as well as eight additional 5S rRNA genes (one each in Scaffold_1 and Scaffold_2, four in Scaffold_3, and two more in Scaffold_5). The rest of the contigs were analyzed with the ARAGORN server (9) to identify 78 tRNA genes. The 16S rRNA gene sequence (1,742 bp) reported here shows that this strain is closely related to several Escherichia coli strains. The 4,071 ORFs predicted by the RAST server will be useful for predicting genes involved in antibiotic resistance, plasmid conjugation, transfer of genes, etc., and for understanding the phenotypic characteristics observed in this bacterium.

### Data availability.

This whole-genome shotgun project has been deposited at DDBJ/ENA/GenBank under the accession number QKQU00000000. The version described in this paper is version QKQU01000000. The Sequence Read Archive (SRA) accession number is SRR7824862.
